# Annotations

**Published:** 1908-04-11

**Authors:** 


### ANNOTATIONS.

### Enzymes in Bananas.

When a fruit such as the banana becomes ripe, and still more when it reaches the pulpy stage of over-ripeness, it might well be supposed that micro-organisms are at work, and that very likely the over-ripe fruit might be harmful upon that account. This is not the case, however. Dr. Giuseppe Tallarico has done some very elaborate and exhaustive work upon the subject, publishing his full papers upon "Gli Enzimi ideolitici e catalizzanti nel processo di maturazione delle frutta" in the "Archivio di Farmacologia sperimentale e Scienze affini." His main conclusions are twofold: first, that the pulp of the banana remains absolutely free from microbes so long as the pericarp is intact; cultivations upon bread, agar, gelatine, and so forth remained completely sterile. Secondly, that the maturation of the fruit is due to ferments, of which there are three main kinds—amylotic, invertive, and proteolytic—each of which is present in quantity in the ripe banana. It is, perhaps, upon this account that the fruit is so beneficial in many cases of simple dyspepsia.

### Insurance against Actions for Negligence.

In the issue of *Truth* dated April 1 an interesting suggestion made by a correspondent, a London solicitor, is discussed. He asks why professional men should not be able to insure against accidents due to negligence in their profession, just as business men can insure against similar trade risks. He believes that professional men would be only too glad to avail themselves of such a policy at a moderate premium. The editor, commenting upon this proposition, suggests that Lloyds might quite conceivably be induced to issue policies upon these lines, and that the ordinary insurance companies would probably soon follow suit. The editor of *Truth* further remarks that, in his opinion, doctors would be found even more ready to insure against their liability for professional negligence, since they are very much more subject to the risk of such actions, and the claims that are made against them have generally much less foundation than in the case of solicitors. We quite agree with the editor in his estimate of the relative frequency with which actions for negligence are brought against members of the medical profession, and we appreciate his frank recognition of the baseless nature of the majority of such claims. At first sight it would seem that the suggestion of small annual policies against these risks with respectable companies should commend itself to medical men; but before going any further with the subject it would be well to remember that membership of one of the large Medical Defence and Protection Societies is in itself to all intents and purposes an insurance against unjust, fraudulent, and malicious claims in respect of alleged professional negligence or misconduct. These societies do not, of course, recompense their members in the event of the patients' claims being upheld by the Courts of Justice; but by prompt, energetic, and whole-hearted action they so frequently overthrow such claims (often before legal proceedings have actually been begun) that their clients' risks of wrongful action for negligence are almost eliminated. This being the case, there is some doubt in our minds as to the present need for such an insurance scheme as is proposed. A recent important trial, as the result of which, in spite of the help of a powerful society and the moral support of the bulk of the profession, two Welsh surgeons were heavily penalised, seems too exceptional in many respects to justify direct insurance against actions for professional negligence.

### Day Nurseries.

A social observer who had not on the whole a high opinion of the London workman—especially the kind that is often out of work—recently felt bound to add, "but the women are splendid." A large number of the working women of London are married; their labour as charwomen, laundresses, sack-makers, flower-makers, and all the innumerable other trades they take up has in many cases to support entirely themselves and their children, and perhaps an invalid or unemployed or drunken husband as well. How nobly and unselfishly they do it those who go in and out among them know. But if they go out to work they must leave their children. Where? Too often in the streets, where they are in the way of dangers of every kind, or shut up at home. It is such a state of things as this that has induced the establishment of day nurseries in several places. The day nursery is a charity to which it is impossible to object. It is not pauperising. The mothers pay for the care-taking and feeding of their children. It is only a few pence a day—3d. per child is a common charge, but in some places it is 4d. for a single child and two for 6d.—but even this makes a considerable hole in the earnings of the average woman. It would be outrageous to ask them to pay more. But of course, though this sum may, with good management, meet the cost of the children's meals, it cannot meet the cost of rent, taxes, firing, and nurses' wages. That must come from the charity of the richer classes of the community. In many places sufficient local contributions are available, but unfortunately the very spots where day nurseries are most needed are those where the bulk of the inhabitants are poor. Such is Hoxton, and an appeal for funds to maintain day nurseries there should find support far outside the grimy and poverty-stricken limits of the neighbourhood. A subscription made to these day nurseries is a gift made to the children of the district, giving them the chance of better food and better daily care than their mothers can, under existing circumstances, afford them; giving them, therefore, a better chance of growing up to that health of body and brightness of mind by which they may hope—and we for their sakes—to rise to a better and less helpless position than their parents. The hon. treasurer of the day nurseries is the Hon. Claude Hay, M.P., and the head office is at 376 Strand.

